# Implementation of structured feedback in a psychiatry residency program in Canada: a qualitative analysis study

**DOI:** 10.3389/fpsyt.2023.1276985

**Published:** 2023-11-13

**Authors:** Anupam Thakur, Shaheen Darani, Csilla Kalocsai, Ivan Silver, Sanjeev Sockalingam, Sophie Soklaridis

**Affiliations:** ^1^Centre for Addiction and Mental Health, Toronto, ON, Canada; ^2^Department of Psychiatry, University of Toronto, Toronto, ON, Canada; ^3^Sunnybrook Research Institute, Toronto, ON, Canada

**Keywords:** R2C2 feedback, supervisor, intersectionality, implementation, CFIR, psychiatry, residency

## Abstract

**Introduction:**

Structured feedback is important to support learner progression in competency-based medical education (CBME). R2C2 is an evidence-based four-phased feedback model that has been studied in a range of learner contexts; however, data on factors influencing implementation of this model are lacking. This pilot study describes implementation of the R2C2 model in a psychiatry CBME residency program, using the Consolidated Framework for Implementation Research (CFIR).

**Methods:**

The study was carried out in three phases: planning, implementation and evaluation. After receiving training, 15 supervisors used the R2C2 feedback model with residents. Semi-structured interviews explored (*n* = 10) supervisors’ experience of the model. CFIR was used to identify factors that influence implementation of the R2C2 model when providing feedback to residents.

**Results:**

Qualitative data analysis revealed four key themes: Perceptions about the R2C2 model, Facilitators and barriers to its implementation, Fidelity to R2C2 model and Intersectionality related to the feedback. The CFIR implementation domains provided structure to the themes and subthemes.

**Conclusion:**

The R2C2 model is a helpful tool to provide structured feedback. Structure of the model, self-efficacy, in-house educational expertise, learning culture, organizational readiness, and training support are important facilitators of implementation. Further studies are needed to explore the learner’s perspective and fidelity of this model.

## Introduction

Feedback is crucial to residents’ progress in Competency-Based Medical Education (CBME) (1). Studies demonstrate that feedback in clinical settings often lack necessary resources to support a progressive learner experience (2–4). As post-graduate programs move toward the adoption of CBME, the need for providing feedback to learners has become increasingly important. Implementation of CBME can be supported by structured, deliberate feedback models for learners (4, 5). Various models of feedback for learners such as the “sandwich model” (6) and “Ask-Tell-Ask” (7) model have been described in literature. Within a CBME framework, the later model provides opportunities for learner’s self-assessment, observer’s assessment and develop an action plan to address issues raised in the session. This allows trainees to develop self-assessment skills but lacks coaching for change. Feedback models that facilitate a coaching relationship with the supervisor can help with learner’s progress and outcomes (8). Further, regulatory bodies have emphasized on the need for faculty members to support learners by engaging in a feedback model that is aligned to the principles of coaching, assuming the role of a “feedback coach” (9). Within medical education domain, coaching is identified as an educational philosophy to support learners’ professional and personal growth and help them achieve their potential (9).

The R2C2 (relationship, reaction, content, coaching) model of feedback fits well within the CBME framework as it provides a structured approach to both feedback and coaching for medical learners. This model draws on strong theoretical foundations of person-centeredness, informed self-assessment and science of behavior change (4). It consists of four phases: relationship building, exploring reactions to the feedback, exploring understanding of feedback content, and coaching for performance change. The role of R2C2 in feedback for physicians and residents has been explored in several studies. Table 1 provides a brief review of studies using R2C2 as a feedback tool (4, 10–15). The model is useful in both longitudinal and in-the-moment feedback (15). More recent literature also points to the importance of “coaching conversations” (14) in facilitating clinical competencies, which is a crucial part of this model. Existing research has identified factors influencing R2C2 implementation include feedback-provider engagement (4, 13), support to feedback providers, organizational support, faculty development, and program evaluation. However, studies on feedback to date have not used a conceptual framework to examine the factors influencing application of such models.

**Table 1:** Review of studies using R2C2 as a feedback model.

Authors, Year, Country	Participants	Study methods	Key findings
Sargeant et al. (2015) (10), USA, Canada	Modeling (*n* = 6) Feasibility pilot (2 physician facilitators, 4 physician recipients) Facilitator preparation (*n* = 8) Model feasibility testing (8 physician facilitators, 8 physician recipients) Multiple specialties	Objectives: development and feasibility testing of R2C2 facilitated feedback model Multistage, qualitative study focused on modeling, facilitator preparation, model feasibility testing, model refinement Model development approach (theoretical framework): person-centered, informed self-assessment, behavioral change principles Feasibility testing involved acceptability, compliance, stability of the intervention, consistency of delivery, recruitment and retention, influence of varying contexts	Four phases of R2C2 feedback: build relationship, explore reactions, explore content, coach for performance change The model was acceptable and stable across different physician contexts Each phase drew on one or more of the theoretical frameworks used in developing the model Small sample size, voluntary participation, time commitment of facilitators and recipients were described as limitations
Sargeant et al. (2017) (11), Canada	5 supervisors, 7 residents Two specialties	Objective: explore the utility and acceptability of the R2C2 model in residency education Supervisor preparation – either 1 h workshop or 1:1 training (orientation to evidence and theory, explanation of model, practice using the model, trifold brochure provided) Qualitative study, feedback transcripts were analyzed	Study demonstrated engagement of supervisors and residents in the feedback R2C2 was seen as a reflective model consistent with competency-focused medical education models Small sample size, need to include more residency programs was highlighted
Sargeant et al. (2018) (4), Canada, USA, the Netherlands	21 supervisors, 45 residents Multiple specialties	Objectives: explore the effectiveness of the R2C2 feedback model and factors influencing effectiveness Two feedback sessions with each resident three to six months apart In three stages: preparation (site assessment), model testing (educational intervention for supervisors), and model refinement (evaluation of model to guide revisions) Criteria for evaluation of effectiveness: resident’s reflection and engagement with feedback, identification of opportunities for improvement, learning change plan (LCP)	Factors influencing effectiveness: resident-supervisor relationship, commitment and engagement of supervisors, resident’s familiarity with reviewing their own data, program assessment approaches across sites, program culture and context of feedback, supports for implementation (preparatory workshop, R2C2 brochures, LCP), presence of an author/research associate during implementation
Lockyer et al. (2020), USA, Canada	11 supervisors Multiple specialties	“In-the-moment” feedback adaptation Co-creation of action plan Used longitudinally as well as for one or more shifts Settings included outpatient clinics, operating rooms and inpatient units	R2C2 can be used for shorter, more frequent conversations All 4 phases important in the shorter format Specific communication and facilitation strategies needed to engage the learner
Graham and Beuthin (2018) (12), Canada	Nurse practitioners (*n* = 12)	Exploratory study using R2C2 used as a coaching tool along with online multi-source feedback (intervention) Post-intervention survey and repeated after 2 months	Participants described the intervention as helpful 63% participants reported a change of practice due to their participation
Armson et al. (2019) (13), Canada, USA, the Netherlands	15 supervisor-resident dyads Multiple specialties	Study examined process-oriented and content-oriented coaching skills used in coaching session Two audio-taped feedback sessions four months apart, qualitative analysis of feedback process and content	Process skills: preparation, relationship development, using “micro communication skills,” reflection and self-assessment by the resident, supervisor flexibility. Content skills: engagement, collaborative discussion, goal setting, co-developing a plan Limitations: 15 dyads, variability in supervisors’ approach to coaching
Parson et al. (2020) (14), USA	12 students, 2 faculty mentors (pilot) 36 students, 6 mentors in subsequent years Pre-clerkship, 18 months, longitudinal	Objectives: promote identity formation, clinical competency Use of “coaching conversations,” individualized formative feedback and goal setting Longitudinal coach-learner relationship Meeting preparation: students and faculty coaches review data from clinical assessments including the results of Entrustable Professional Activity (EPA) assessments and reports, clinical evaluations, self-reflection of students, learning plans Meeting: Co-creation of learning goals and co-construction of action plan	Pilot with smaller group followed by broader roll-out (feasibility) Cultivating a coaching culture (monthly faculty development sessions, quarterly large group retreats, regular meetings for coaches) important for implementation Institutional support was vital: developing a coaching role; 30% FTE for coaching Need for program evaluation and evaluation of the coaches was highlighted

**Figure 1:**
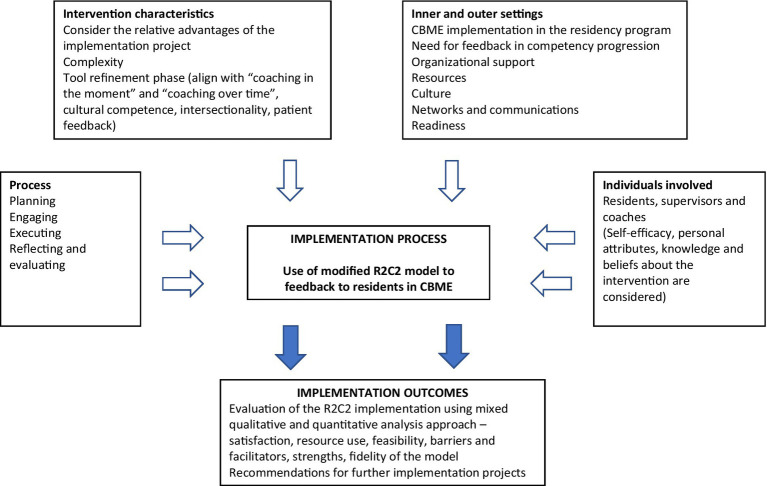
CFIR framework for R2C2 model of feedback.

Implementation science frameworks provide a valuable approach for medical educators and researchers to integrate best evidence into education practice (16). It provides structures to understand changes in learner outcomes, such as competence and performance that occur within a given context, and are useful when considering generalizability of approaches and findings (17). The Consolidated Framework for Implementation Research (CFIR) is an implementation science approach that is used to facilitate the design, evaluation, and implementation of best evidence into practice (18). It is an useful conceptual framework to guide and evaluate the implementation process and identify influential factors of interventions across healthcare (17–20); and in medical education settings (21–23). The main domains in CFIR are *intervention characteristics (evidence, strength, quality of the R2C2 model)*, *inner and outer settings (organizational support, resources, readiness)*, *individual characteristics (self-efficacy, knowledge and belief of supervisors and residents about the R2C2 feedback model)*, and *process (planning, engagement, reflection and evaluation)* of implementation (24). CFIR provides a menu of constructs arranged across various domains (Figure 1). This study adds to the literature that tries to bring implementation science approaches into program evaluation research in medical education.

The aim of the pilot study was to describe the implementation and the factors that influence implementation of the R2C2 model of feedback in a psychiatry CBME residency program, using this framework. In response to the gender and racial biases shown in the literature (25–28) and a growing concern about equity, diversity and inclusion within medical education (28–30), we introduced faculty to the concept of intersectionality during the training and evaluation phases, with a longer-term goal of incorporating intersectionality into the R2C2 model. Given the importance of this topic, power dynamics in the feedback process has been critically evaluated in a separate paper.

## Materials and methods

### Setting

This pilot study was conducted at the Centre for Addiction and Mental Health (CAMH) between July 2019 and November 2020. CAMH employs approximately 250 physicians and hosts approximately 180 postgraduate trainees annually. Study participants included supervisors of residents, postgraduate year 1 (PGY1) to fourth year of residency training, in the Competence by Design (CBD) stream of the University of Toronto Psychiatry Residency Program at CAMH. Royal College of Physicians and Surgeons of Canada (RCPSC) has supported the implementation of Competence by Design (CBD) system across Canadian residency programs (31). In CBD, training is organized into stages and includes frequent formative assessments of learner capabilities. The CBD stream residents were on rotation in the Longitudinal Ambulatory Experience (LAE), a longitudinal rotation where psychiatry residents, over 3 years of residency training, followed outpatients over their illness course. The primary research question for this study was, *what are the factors influencing implementation of R2C2 feedback model using CFIR framework*? This study was approved in February 2020 by the Quality Projects Ethics Review at CAMH, Toronto.

### Study phases

The pilot study consisted of three phases: planning, implementation, and evaluation.

#### Planning

Prior to applying for funding, with content experts, social scientists and education experts, AT and SD concluded that the CFIR framework would be the most suitable for the study (Figure 1). The CFIR (20) was used to guide systematic assessment of the implementation and to identify factors that influence intervention, implementation and effectiveness. One of the CFIR domains is “intervention characteristics,” which includes intervention source, evidence supporting desirable outcomes, strengths, adaptability, trialability to test the intervention on a small scale, complexity of the implementation process, design considerations and costs involved. During subsequent meetings, the authors of this paper reviewed feedback models using intervention characteristics described in CFIR and chose the R2C2 model because it was theory- and evidence-based and had been adapted and studied in a variety of settings (4, 11, 13, 15, 32). Further, two of the authors (IS, SS) were content experts in R2C2 tool development.

After receiving funding and institutional approval, SS and IS delivered two training sessions (in person in January 2020 and virtual in June 2020) on the R2C2 model. The session in June 2020 was carried out virtually (videoconference) due to COVID-19 pandemic restrictions. A video recording of the training session was available to the supervisors for reference. Training sessions introduced the supervisors to the original R2C2 model (4). In addition, they highlighted the racial and gender bias that may disadvantage racialized students or trainees and introduced the concept of intersectionality through didactic, reflective exercises and small group discussions. Supervisors were provided with additional resources to complement their learning of intersectionality from the training sessions. One resource included a brief summary of phrases and strategies for supervisors to use with residents to start the conversation and reflect on power and privilege within each stage of the R2C2 model (see Appendix 1). The other document included a list of anti-Black racism mental health resources, a trifold leaflet of the R2C2 model and its accompanying phrases and stages (10), and a peer-reviewed article on racial bias in feedback practices experienced by racialized students (33).

#### Implementation

The implementation phase was carried out over 3 months after the training. Following training, the supervisors were offered support through periodic email check-ins, ongoing support from LAE education leaders and one-on-one coaching by R2C2 experts in the team, upon request. Supervisors were encouraged to use the R2C2 model when providing feedback to residents on their clinical encounters with patients, taking intersectionality into consideration.

#### Evaluation

Supervisors in the study were invited to participate in one-on-one semi-structured interviews to share their experience of using the feedback model. We used qualitative research methods to conduct this evaluation, because an in-depth understanding of experience can help us refine the implementation (34).

### Sampling and recruitment

Participants were identified using a purposive sampling approach (34). The project co-leads (AT, SD), through meetings with education leaders, decided that supervisors in the LAE rotation would comprise the sample population. The LAE allowed supervisors to maintain a close connection with their residents over a long term, allowing for ample time for supervisors to apply the R2C2 model to their feedback sessions. There was a total of 15 supervisor-resident dyads in the LAE rotation at CAMH. The Research Analyst (RA) invited, via email, the 15 supervisors that were supervising residents on their LAE rotation to participate in a voluntary interview about their experience using the R2C2 model. One month later, a second reminder invitation was sent to supervisors. Of the 15 supervisors, 10 responded and participated in the interviews, evenly distributed between men and women. No other demographic information was recorded to ensure anonymity of the participants.

### Data collection

Interviews were conducted between August and November 2020 by telephone or videoconference, depending on the preference of the participant. Verbal consent was obtained at the beginning of each interview. The semi-structured interviews consisted of five questions that aimed to elicit participants’ experiences of using R2C2, the implementation process, and their reflections on power dynamics and feedback practices with residents (see Appendix 2 for interview questions). Additional probes were used where necessary for participants to comment on the impact of gender, race or other social categories in the supervisor-resident relationship.

### Data analysis

Interviews were audio-recorded and transcribed verbatim by a professional transcriber and thematically analyzed (35). The CFIR was used as a conceptual framework to inform our coding. We used the domains of the CFIR to organize and deductively analyze the participants’ narratives about the implementation and use of the R2C2 model.

### Reflexivity

The research team met periodically to discuss the coded data and how our individual experiences, values, beliefs and identities may influence our reflections and outcomes of the study. We are a diverse group consisting of education leaders, researchers, and clinicians, with varying experiences and that may have influenced the way the study was conceptualized, analyzed and reported.

## Results

Four themes were generated from the interview data: Perceptions about the R2C2 model, Facilitators and barriers to its implementation, Fidelity to R2C2 model and Intersectionality related to the feedback. Given the focus of the study on R2C2 implementation, CFIR implementation domains (20) (in italics) have been used to explain the themes and subthemes (Table 2).

**Table 2:** Themes and sub-themes and corresponding CFIR domains.

Themes	CFIR domains
Perceptions about R2C2 Subtheme: utility of R2C2	Intervention Characteristics (evidence, strength and quality relative advantage)
	Inner Setting (implementation climate)
	Characteristics of Individuals (knowledge and beliefs about the intervention)
Facilitators and barriers to implementation	Intervention Characteristics (design quality, complexity)
	Process of implementation
	Outer Setting (cosmopolitanism)
	Inner setting (culture, reflection and evaluation)
Training recommendations	Inner setting (implementation climate, learning climate, engaging)
	Process of implementation
Fidelity to R2C2 model	Characteristics of individuals (self-efficacy)
Intersectionality	Characteristics of individuals (Other personal attributes)

### Theme 1: perceptions about R2C2

Most supervisors identified the R2C2 model as having important *intervention characteristics*. The majority of participants agreed that there was an advantage to having the model to guide constructive feedback to residents. Most supervisors also highlighted the flexibility and ease of using the model, which allows for non-linear movement between stages of the model. The model made the feedback process more deliberate:

I can see the rationale, the benefit from the model, and its usefulness, and just bringing awareness in certain areas of the feedback process (Participant #4).

Several supervisors also noted the importance of design quality and packaging as an important *intervention characteristic*. As a resource, supervisors were encouraged to consult a trifold leaflet distributed during the planning phase that outlined the stages of the R2C2 model and sample phrases to use with their residents in each stage (7). The trifold resource was deemed as helpful resource to supervisors:

I think that [the trifold leaflet] is easy to review, and there’s lots of examples of sample phrases, so that’s helpful… I find it easy to implement in that regard (Participant #1).

### Theme 2: facilitators and barriers to implementation

All participants identified various factors related to implementation, *inner and outer settings* in particular, which helped in its uptake, and suggested ways to overcome barriers. The *outer setting* of CFIR (24) is characterized by needs, resources and networks with external organizations to support the intervention and emerged as a facilitator for implementation. Several participants commented on the expertise of local faculty leads and their work with external lead researchers across the country in developing the R2C2 model. The *inner setting* of CFIR includes structural characteristics such as availability of faculty members, administrative support to facilitate innovation, learning climate, resource availability, culture and readiness of the organization. Participants appreciated the availability of in-house education experts to conduct the R2C2 training, which increased their confidence and engagement with the model. The culture of a department was integral to the uptake of R2C2 by supervisors:

I think what’s helpful is that there’s a lot of experts in the faculty and the department on this area, and that it’s supported and emphasized. I mean, the culture of any department will either reinforce these kinds of things or not (Participant #4).

Most participants discussed the norms and values of an organization in creating a culture conducive to professional development. Participants described how CAMH education leadership provided a safe learning climate to implement the model. A few participants described how the model provided a helpful structure to follow particularly with residents who were perceived as “fragile” or “challenging”:

This resident was not as receptive to my feedback and what I would do with the R2C2 model is apply some of those more concrete strategies…it was more formalizing or giving me additional strategies to use with a more difficult…a more challenging resident (Participant #8).

On the other hand, the model could overly formalize the feedback process:

It takes some of the art out of it for me of just being present with my students and listening and being aware and attuned to them…. I don’t think it always helps to put really formalized language and steps to it. I think that feels just too mechanical (Participant #10).

Participants reflected on ways to improve the program and shared training recommendations, which mapped to “*process*” and “*inner setting*” in CFIR. Suggested improvements encompass planning, engaging teams (faculty), executing the implementation plan and evaluation. Several participants provided ideas for future faculty development on the model, for example, by incorporating a variety of teaching modalities, including role play, debate, and videos.

Within the *inner setting* perspective, there were no extrinsic incentives during the planning or implementation phases for supervisors to use the model. Thus, incentivizing participants in the study to implement the model was sometimes challenging and mapped to the “facilitators and barriers to implementation” theme.

### Theme 3: fidelity to the R2C2 model

Broadly, implementation fidelity refers to the degree to which an intervention delivers what it intended to deliver. This theme aligned with the *individual characteristics* domain of CFIR framework. Several *individual characteristics* influenced implementation of the R2C2 model including knowledge, attitude, self-efficacy of the faculty, motivation, competence and capacity.

Overall, most participants made reference to using one or more stages of R2C2 model. Participants most often referenced the first stage, “build rapport and relationship” and placed value on developing relationships with their residents that would best support their learning:

I’ve incorporated the R2C2 by initially spending, generally the first session or the first resident, getting to know them. So, building the rapport and figuring out background information, what they hope to do, what they’re interested in (Participant #3).

### Theme 4: intersectionality related to the feedback

Damschroder et al. (24) describes individuals as “carriers of cultural, organizational, professional and individual mindsets.” Individuals can use power and influence on others that can have consequences for implementation. The concept of intersectionality mapped most closely to CFIR’s *individual characteristics.* Although the theory of intersectionality describes the structural or system-level forces that produce inequities, the majority of participants focused on the power dynamics at the individual level, in general, and of the in-the-moment feedback process, in particular.

Some participants felt that reflection on power and positionality were important considerations in the supervisor-resident relationship to feedback. A near equal number of participants believed that gender, race, or other social categories were not relevant to the feedback relationship. One participant shared that he did not have much exposure to racialized communities growing up in his hometown. As he witnessed the various initiatives on equity and diversity at the organization-level, he recognized the need to reflect on his own identity and how it might influence his relationship with residents:

One thing that comes to mind is more about gender and ethnicity. In my first year, I supervised two PGY1 residents. I’m a white male, and one of the residents was a black male, and one of the residents was a white female… I found myself having to reflect more on how to give feedback in a way that would be fair or make sense to them (Participant #2).

Not all participants saw the relevance of considering the concept of intersectionality when providing feedback:

I think most of the residents I work with are female, I just don’t see it as being relevant to the relationship we have (Participant #1).

Additionally, a few participants stated that bringing up power, gender, or race into the relationship was not something they should do:

It’s not something you bring up directly with the residents… it might even be a bit of an uncomfortable conversation to bring up because it’s something that’s assumed as far as the relationship between supervisor and resident goes. And I don’t want to make implications that there’s any issue in terms of gender or race or anything like that (Participant #7).

## Discussion

This pilot study describes the feasibility and factors influencing implementation of the R2C2 model as a feedback model within a LAE psychiatry residency program. Several factors influenced the implementation of the model. For example, supervisors valued the structure and format of the model and the accompanying tools, the flexibility and non-linearity between the phases and its relevance in CBME. The focus on relationship-building and coaching in the model was particularly helpful to supervisors in the longitudinal ambulatory rotation where the supervisor-trainee relationship continues over an extended period of time. Feedback from supervisors in the study suggests that the R2C2 model can be an important tool to develop this educational relationship with trainees. We believe this is the first study that uses an implementation framework for introducing a structured feedback model for supervisors in psychiatry residency training.

We found CFIR to be a useful framework for describing the implementation of the R2C2 model. We were able to map the evaluation data from participants into the various implementation domains of CFIR. The use of an implementation framework in educational interventions is relatively new (17). Our study validates the benefits of using such a tool. Although all the domains were important for implementation (Table 2), the data suggests “*intervention characteristics”* and *“individual characteristics,”* including self-efficacy of the supervisors, were crucial to implementation of the model. Content expertise in the model facilitated its implementation. Additionally, faculty development sessions, organizational readiness, availability of content experts for tool refinement and time spent in planning and engagement were important facilitators in the successful implementation of the R2C2 model. Fidelity of the model and learning culture of the organization were important contributors too.

Although most participants described the model as useful in feedback sessions, some found its inherent structure inflexible. Other participants believed that incentives to implement the model are necessary. Previous studies have identified the need for compensation for quality supervision (14) and time commitment for supervisors (10). Moreover, literature on using implementation science models to evaluate complex education programs and innovations suggests that individuals are likely to engage with and embrace change if it fosters a sense of agency (22). Therefore, it is possible that some supervisors may have found the R2C2 model as being too rigid, thus limiting supervisors’ perceived agency in the feedback process.

Several participants found that a structured and evidence-based approach to feedback provided a safe space for both supervisors and residents. Many participants were comforted to know that they were following a model as opposed to providing feedback in a more informal, undocumented or *ad hoc* manner. The scaffolding of safety in a structured model is important in the context of intersectionality as a previous study has shown that individuals from racialized backgrounds perceive negative bias in receiving constructive feedback (36). Not surprising, the participants in this study were divided about incorporating or discussing power and inequity within the feedback relationships with residents. The process of change itself, including early and later adopters can explain this variance (37, 38). Access to further training in equity, diversity and power imbalances can help supervisors with understanding these concepts while incorporating them in learning conversations. Through our pilot, it became clear that more training on power dynamics and the concept of intersectionality in the feedback process was needed to fully implement such concepts into the ethos and practice of providing feedback. In addition, it was noted that more work needs to be done to move the concepts of power and inequality from being considered as individual characteristics to recognizing them as systemic and structural forces. Our study provided an introduction to these concepts and how they might be used to address gender and racial biases in feedback processes described in the literature (27, 39, 40). As such, study findings showed that supervisors were ambivalent about the value of considering power dynamics and intersectionality in the process of providing feedback. These findings suggest the need to explore the impact of intersectionality and power dynamics on feedback processes in the supervisor-resident relationship.

COVID-19 has had significant economic, social, educational and mental health impact on the society globally (41). Lockdowns, quarantines and other isolation measures propelled innovations in the health system to provide health care as well support the educational needs of learners. Just as virtual care has rapidly scaled up to meet the challenges in clinical care due to the pandemic, medical education too has stepped up to the challenge. We pivoted the implementation of the feedback model to a virtual format due to the COVID-19 pandemic. Rapid innovations played a key role in moving to a virtual mode (42). Telephone consultation support ensured that participants were able to make the transition to a virtual R2C2 model seamlessly. The ability to implement this model to provide virtual feedback during COVID-19 suggests that the R2C2 model implementation was adaptable and responsive to changing system needs, which has been described as a key component to successful implementation of new education program changes. Nonetheless, participants identified several strategies for preparing supervisors for providing feedback virtually including the use of role plays and videos to train supervisors on this virtual feedback model. Very few studies have explored the role of structured feedback models such as R2C2 in virtual sessions; this warrants future research.

Our study has a number of limitations. The evaluation plan could have been more robust by including the resident experience or impact on the learner. This study was also limited by the focus on a single specialty area in medicine and a small sample size. The timing of the interviews may have influenced the views and convictions of the participants about the R2C2 model and its implementation. Whether longer implementation phase would have led to different views from participants is not known. Further, this study took place in a single institution that has prioritized medical education and faculty development to support supervisors in their teaching practices.

Future directions for research include exploring the impact on the learner and experience of residents that receive feedback from supervisors using the R2C2 model both longitudinally as well as in-the-moment feedback. We started a conversation with supervisors on the concept of intersectionality and feedback. More research is needed to better understand the divided views that emerged during the interviews. Future studies comparing R2C2 model to existing feedback system and including residents who provide feedback can be useful. Supervisor characteristics which can influence implementation including supervision experience and familiarity with structured tools need further exploration. The study was conducted virtually for the most part due to the pandemic. Its generalizability and validity in the post-pandemic era need to be examined. The perspective of trainees, the actual quality of the feedback conversation and the sustainability of such a change implementation need to be explored in future studies. Also, studies should include fidelity measures to understand the use of the model with intersectionality training. Faculty development that incorporates a variety of teaching modalities and highlights the importance of intersectionality in feedback conversations will be important for future implementation efforts, including large-scale implementation.

## Conclusion

1. R2C2 model of feedback is a helpful tool to provide structured feedback.
2. Implementation frameworks such as CFIR can be helpful in implementation of feedback models in residency programs.
3. Structure of the model, self-efficacy of users, learning culture and training support are some of the important facilitators of implementation.
4. Further studies are needed to explore the learner’s perspective and fidelity of this model.

## Data availability statement

The raw data supporting the conclusions of this article will be made available by the authors, without undue reservation.

## Ethics statement

The studies involving humans were approved by Centre for Addiction and Mental Health Quality Projects Ethics Review. The studies were conducted in accordance with the local legislation and institutional requirements. Written informed consent for participation was not required from the participants or the participants’ legal guardians/next of kin because verbal consent was obtained from participants. This was approved by CAMH Quality Projects Ethics Review.

## Author contributions

AT: Conceptualization, Data curation, Formal analysis, Funding acquisition, Investigation, Methodology, Project administration, Visualization, Writing – original draft, Writing – review & editing. SD: Conceptualization, Data curation, Formal analysis, Funding acquisition, Investigation, Methodology, Project administration, Visualization, Writing – original draft, Writing – review & editing. CK: Methodology, Writing – original draft, Writing – review & editing. IS: Conceptualization, Supervision, Writing – original draft, Writing – review & editing. SaS: Conceptualization, Funding acquisition, Supervision, Writing – original draft, Writing – review & editing. SoS: Conceptualization, Data curation, Formal analysis, Funding acquisition, Investigation, Methodology, Supervision, Writing – original draft, Writing – review & editing.
