# Supplementary material for: Implementation of structured feedback in a psychiatry residency program in Canada: a qualitative analysis study
Source: Front Psychiatry. 2023 Nov 13;14:1276985. doi: 10.3389/fpsyt.2023.1276985 (PMC10699149; doi:10.3389/fpsyt.2023.1276985)
Supplement: Supplementary file 1 [file Data_Sheet_1.pdf]

Stage 1 Strategies and sample phrases

(before the patient is brought in)

- *"How has the rotation gone for you? What did you enjoy, what challenged you about it?"*
- *"Tell me about your feedback experiences on Mock orals to date. What's been helpful and what hasn't?"*
- *"How do you think you're doing? What are your strengths and opportunities to improve?"*
- *"What would you hope to get out of this feedback discussion?"*

Confirm what you're hearing; show respect; build trust; validate.

Relationship-building is central and needs attention throughout all Stages of the Feedback process.

Stage 2 Strategies and sample phrases

(after the interview and presentation)

- *"What were your initial reactions? Anything particularly striking?"*
- *"Did anything in the feedback surprise you? Tell me more about that..."*
- *"How do this feedback compare with how you think you were doing? Any surprises?"*

Negative reactions/surprises tend to be more frequently elicited by:

- Feedback identifying one is not doing as well as they thought.

Be prepared for expression of negative reactions in these cases. Phrases to validate negative reactions & support:

- *"You're not the first one to identify that as a stumbling block"*
- *"It's difficult to hear feedback that disconfirms how we see ourselves"*
- *"We're all trying to do our best and it's tough to hear when we're not hitting the mark"*
- *"We're going to work together"*

Stage 3 Strategies and sample phrases

(after the interview and presentation)

- *"Let's go through the interview and presentation, section by section."*
- *"Is there anything in the feedback that doesn't make sense to you?"*
- *"Anything you're unclear about?"*
- *"Anything in section X that you'd like to explore further or comment on?"*
- *"Anything that struck you as something to focus on?"*
- *"Do you recognize a pattern?"*

A careful review of the interview and presentation skills and identification of performance gaps will guide Stage 4, Coaching.

Stage 4 Strategies and sample phrases

(after the interview and presentation)

Before developing a learning/ change plan, residents need to understand and accept the content of their feedback.

Consider coaching as:

- guiding the development of goals and activities to achieve them
- supporting self-directed learning
- the "skill of offering solutions."
- ensuring a concrete plan is developed

- *"What do you see as the prioritie/s for your improvement?"*
- *"What would you like to achieve for your next Mock oral?"*
- *"What 1-3 things would you target for immediate action?"*
- *"What would be your goal for this?"*
- *"What actions will you have to take?"*
- *"Who/what might help you with this change?"*
- *"What might get in the way?"*
- *"What else might you do to progress to the next level?"*
- *"Do you think you can achieve it?"*
