# Supplementary material for: Implementation of structured feedback in a psychiatry residency program in Canada: a qualitative analysis study
Source: Front Psychiatry. 2023 Nov 13;14:1276985. doi: 10.3389/fpsyt.2023.1276985 (PMC10699149; doi:10.3389/fpsyt.2023.1276985)
Supplement: Supplementary file 2 [file Data_Sheet_2.pdf]

Modified R2C2 Interview Guide for Supervisors

The following are examples of interview questions:

1. How did you incorporate the R2C2 model and its modified form in your feedback practices? Please provide as much detail as possible.
    a. Could you provide us some concrete examples from your meetings with the residents?
2. How do you think the modifications influenced your feedback practices, especially since the modifications encourage supervisors to pay attention to power differences that exist between supervisors and residents?
    a. Probe: How have you paid attention to the power differences that exist between you and your residents (staff-student, race/ethnicity, gender/sexuality and class)? Can you provide feedback experiences that speak to this?
    b. Probe: How do you feel that attention to the residents' gender influenced the feedback, mentorship opportunities and resources you provide? Can you provide feedback experiences that speak to this?
    c. Probe: Do you hesitate when it comes to giving feedback to women because you worry about sounding mean or hurtful? Can you provide feedback experiences that speak to this?
3. What did you find helpful as well as challenging in the interventions?
4. What did you find helpful and challenging in the implementation of these interventions?
5. Would you have any suggestions for improving feedback or the implementation of these interventions?
